# 1,10-Phenanthrolinium 4-chloro-2-hydroxy­benzoate–1,10-phenanthroline–4-chloro-2-hydroxy­benzoic acid (1/1/1)

**DOI:** 10.1107/S1600536808015110

**Published:** 2008-05-24

**Authors:** Hong Shen, Jing-Jing Nie, Duan-Jun Xu

**Affiliations:** a Department of Chemistry, Zhejiang University, People’s Republic of China

## Abstract

The title compound, C_12_H_9_N_2_^+^·C_7_H_4_ClO_3_^−^·C_12_H_8_N_2_·C_7_H_5_ClO_3_, contains one phenanthrolinium (Hphen) cation, one phenanthroline (phen) mol­ecule, one 4-chloro-2-hydroxy­benzoate anion (hcba) and one 4-chloro-2-hydroxy­benzoic acid (Hhcba) mol­ecule in the asymmetric unit. The phen mol­ecule is approximately parallel to Hphen, making a dihedral angle of 1.98 (6)°. The centroid–centroid distance between pyridine rings of adjacent phen and Hphen species is 3.7718 (15) Å, and that between the benzene and pyridine rings of adjacent phen and Hphen species is 3.7922 (16) Å, indicative of π–π stacking inter­actions. The crystal structure contains an extensive network of classical (O—H⋯O, N—H⋯N and O—H⋯Cl) and weak (C—H⋯O and C—H⋯N) hydrogen bonds. Finally, C—H⋯π inter­actions are seen between Hphen and hcba and between phen and Hhcba in the crystal structure. The hydroxy group of the anion is disordered over the two sites *ortho* to the carboxylate group in a 0.75:0.25 ratio.

## Related literature

For general background, see: Su & Xu (2004 ▶); Pan *et al.* (2006 ▶). For a related structure, see: Fu *et al.* (2005 ▶).
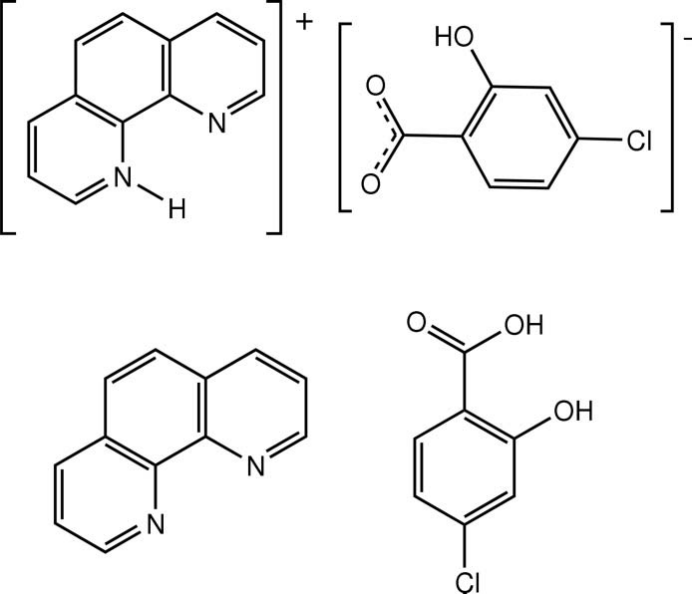


## Experimental

### 

#### Crystal data

C_12_H_9_N_2_^+^·C_7_H_4_ClO_3_^−^·C_12_H_8_N_2_·C_7_H_5_ClO_3_
*M* *_r_* = 705.53
Orthorhombic, 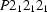

*a* = 8.0627 (6) Å
*b* = 19.6005 (15) Å
*c* = 20.7929 (17) Å
*V* = 3286.0 (4) Å^3^
*Z* = 4
Mo *K*α radiation
μ = 0.25 mm^−1^
*T* = 295 (2) K
0.43 × 0.37 × 0.32 mm

#### Data collection

Rigaku R-AXIS RAPID IP diffractometer
Absorption correction: none
37126 measured reflections
6394 independent reflections
4326 reflections with *I* > 2σ(*I*)
*R*_int_ = 0.054

#### Refinement

*R*[*F* ^2^ > 2σ(*F* ^2^)] = 0.038
*wR*(*F* ^2^) = 0.098
*S* = 0.98
6394 reflections
460 parameters
H-atom parameters constrained
Δρ_max_ = 0.24 e Å^−3^
Δρ_min_ = −0.14 e Å^−3^
Absolute structure: Flack (1983 ▶), 2739 Friedel pairs
Flack parameter: −0.09 (5)

### 

Data collection: *PROCESS-AUTO* (Rigaku, 1998 ▶); cell refinement: *PROCESS-AUTO*; data reduction: *CrystalStructure* (Rigaku/MSC, 2002 ▶); program(s) used to solve structure: *SIR92* (Altomare *et al.*, 1993 ▶); program(s) used to refine structure: *SHELXL97* (Sheldrick, 2008 ▶); molecular graphics: *ORTEP-3 for Windows* (Farrugia, 1997 ▶); software used to prepare material for publication: *WinGX* (Farrugia, 1999 ▶).

## Supplementary Material

Crystal structure: contains datablocks I, global. DOI: 10.1107/S1600536808015110/hb2735sup1.cif

Structure factors: contains datablocks I. DOI: 10.1107/S1600536808015110/hb2735Isup2.hkl

Additional supplementary materials:  crystallographic information; 3D view; checkCIF report

## Figures and Tables

**Table 1:** Hydrogen-bond geometry (Å, °)

*D*—H⋯*A*	*D*—H	H⋯*A*	*D*⋯*A*	*D*—H⋯*A*
O1—H1*A*⋯O5	0.88	1.61	2.484 (2)	173
N1—H1*N*⋯N3^i^	0.85	2.14	2.915 (3)	153
O3—H3*A*⋯Cl1^ii^	0.95	2.66	3.1714 (19)	114
O3—H3*A*⋯O2	0.95	1.86	2.603 (3)	133
O6*A*—H6*A*⋯O4	0.94	1.74	2.584 (3)	148
O6*B*—H6*B*⋯O5	0.82	1.80	2.494 (7)	142
C5—H5⋯O2^iii^	0.93	2.50	3.404 (3)	164
C12—H12⋯O4^ii^	0.93	2.51	3.381 (3)	157
C21—H21⋯N2^i^	0.93	2.51	3.345 (4)	150
C22—H22⋯O1^iv^	0.93	2.54	3.220 (4)	130
C37—H37⋯O4	0.93	2.60	3.430 (3)	150
C25—H25⋯*Cg*1	0.93	2.65	3.571 (3)	174
C40—H40⋯*Cg*2	0.93	2.63	3.489 (3)	154

Symmetry codes: (i) 
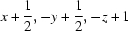
; (ii) 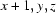; (iii) 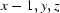; (iv) 
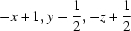
. *Cg*1 is the centroid of the C8–C13 benzene ring and *Cg*2 is the centroid of the C1–C6 benzene ring.

**Fig. 1.**
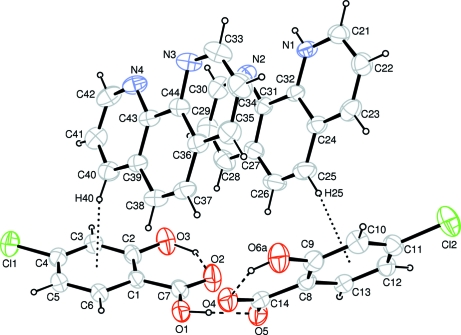
The molecular structure of (I) with 30% probability displacement ellipsoids (arbitrary spheres for H atoms). The minor component of the disordered 4-chloro-2-hydroxybenzoate has been omitted for clarity. Dashed lines indicate hydrogen bonding; dotted lines indicate C—H···π interaction.

**Fig. 2.**
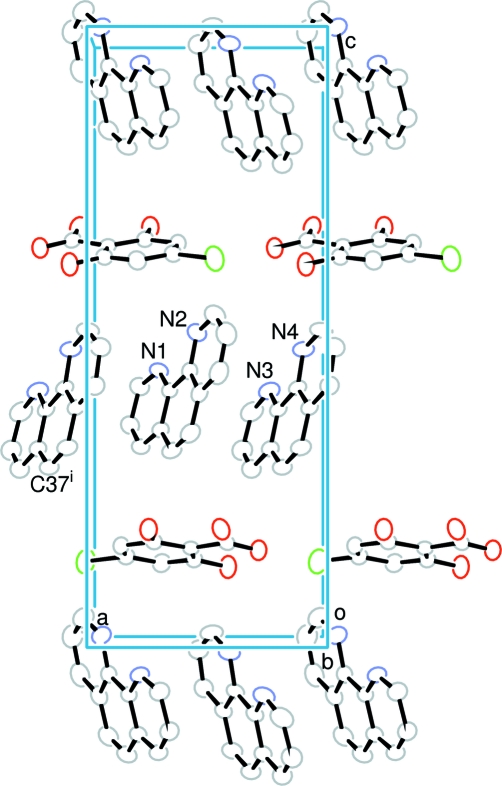
A packing diagram for (I) showing π-π stacking between phen and Hphen ring systems [symmetry code: (i) 1 + x,y,z]. The H atoms are omitted for clarity.

## supplementary crystallographic information

### Comment

As part of our ongoing investigation on the nature of π-π stacking (Su & Xu, 2004; Pan *et al.* 2006), the title compound, (I), incorporating 1,10-phenanthroline (phen) has been prepared and its crystal structure is reported here.

The asymmetric unit of (I) contains two neutral molecules, one cation and one anion (Fig. 1), similar to the situation in 1,10-phenanthrolinium 6-carboxypyridine-2-carboxylate 1,10-phenanthroline pyridine-2,6-dicarboxylic acid (Fu *et al.*, 2005). The significant difference in C-O bond distances [1.312 (3) and 1.239 (3)Å] suggests that the C7-carboxyl group is protonated in the crystal. The neutral 4-chloro-2-hydroxybenzoic acid (Hhcba) is hydrogen bonded with the 4-chloro-2-hydroxybenzoate anion (hcba) (Fig. 1 and Table 2). The neutral phenathroline (phen) molecule is approximately parallel to the protonated phenathroline cation (Hphen), with a dihedral angle of 1.98 (6)°. Fig. 2 shows the nearly parallel arrangement of phen and Hphen. The centroid-to-centroid distances between N2-pyridine and N4-pyridine rings is 3.7718 (15) Å; the centroid-to-centroid distance between N1-pyridine and C37^i^-benzene rings is 3.7922 (16) Å [symmetry code: (i) 1 + *x*,*y*,*z*]. They suggest the existence of π-π stacking between phen and Hphen.

The crystal structure contains C—H···π interactions between Hphen and hcba and between phen and Hhcba (Fig. 1). H25···*Cg*1 = 2.64 Å, C25—H25···*Cg*1 = 174° and C25···*Cg*1 = 3.571 (2) Å (where *Cg*1 is the centroid of the C8-benzene ring); H40···*Cg*2 = 2.63 Å, C40—H40···*Cg*2 = 153° and C40···*Cg*2 = 3.489 (3) Å (where *Cg*2 is the centroid of the C1-benzene ring).

### Experimental

4-chloro-2-hydroxybenzoic acid (0.17 g, 1 mmol) and 1,10-phenanthroline (0.20 g, 1 mmol) were dissolved in ethanol-water (10 ml, 7:3 v/v) at room temperature. The solution was filtered and red-brown chunks of (I) were obtained from the filtrate after 3 d.

### Refinement

The O6-hydroxyl group is disordered over two sites; occupancies were initially refined, and fixed as 0.75:0.25 at final cycles of refinement.  The H atoms bonded to N1 atom and hydroxyl H atoms were located in a difference Fourier map and refined as riding in their as-found relative position, with *U*_iso_(H) = 1.5*U*_eq_(O,*N*). Aromatic H atoms were placed in calculated positions with C—H = 0.93 Å, and refined in riding mode with *U*_iso_(H) = 1.2*U*_eq_(C).

### Crystal data

**Table:**

C_12_H_9_N_2_^+^·C_7_H_4_ClO_3_^–^·C_12_H_8_N_2_·C_7_H_5_ClO_3_	*F*_000_ = 1456
*M**_r_* = 705.53	*D*_x_ = 1.426 Mg m^−^^3^
Orthorhombic, *P*2_1_2_1_2_1_	Mo *K*α radiation λ = 0.71073 Å
Hall symbol: P 2ac 2ab	Cell parameters from 3820 reflections
*a* = 8.0627 (6) Å	θ = 2.0–25.0º
*b* = 19.6005 (15) Å	µ = 0.25 mm^−^^1^
*c* = 20.7929 (17) Å	*T* = 295 (2) K
*V* = 3286.0 (4) Å^3^	Chunk, red brown
*Z* = 4	0.43 × 0.37 × 0.32 mm

### Data collection

**Table:**

Rigaku R-AXIS RAPID IP diffractometer	6394 independent reflections
Radiation source: fine-focus sealed tube	4326 reflections with *I* > 2σ(*I*)
Monochromator: graphite	*R*_int_ = 0.054
Detector resolution: 10.00 pixels mm^-1^	θ_max_ = 26.0º
*T* = 295(2) K	θ_min_ = 1.4º
ω scans	*h* = −9→9
Absorption correction: none	*k* = −24→24
37126 measured reflections	*l* = −23→24

### Refinement

**Table:**

Refinement on *F*^2^	Hydrogen site location: inferred from neighbouring sites
Least-squares matrix: full	H-atom parameters constrained
*R*[*F*^2^ > 2σ(*F*^2^)] = 0.039	*w* = 1/[σ^2^(*F*_o_^2^) + (0.051*P*)^2^] where *P* = (*F*_o_^2^ + 2*F*_c_^2^)/3
*wR*(*F*^2^) = 0.098	(Δ/σ)_max_ < 0.001
*S* = 0.98	Δρ_max_ = 0.24 e Å^−^^3^
6394 reflections	Δρ_min_ = −0.14 e Å^−^^3^
460 parameters	Extinction correction: none
Primary atom site location: structure-invariant direct methods	Absolute structure: Flack (1983), 2739 Friedel pairs
Secondary atom site location: difference Fourier map	Flack parameter: −0.09 (5)

### Special details

**Table:**

Geometry. All e.s.d.'s (except the e.s.d. in the dihedral angle between two l.s. planes) are estimated using the full covariance matrix. The cell e.s.d.'s are taken into account individually in the estimation of e.s.d.'s in distances, angles and torsion angles; correlations between e.s.d.'s in cell parameters are only used when they are defined by crystal symmetry. An approximate (isotropic) treatment of cell e.s.d.'s is used for estimating e.s.d.'s involving l.s. planes.
Refinement. Refinement of *F*^2^ against ALL reflections. The weighted *R*-factor *wR* and goodness of fit *S* are based on *F*^2^, conventional *R*-factors *R* are based on *F*, with *F* set to zero for negative *F*^2^. The threshold expression of *F*^2^ > σ(*F*^2^) is used only for calculating *R*-factors(gt) *etc*. and is not relevant to the choice of reflections for refinement. *R*-factors based on *F*^2^ are statistically about twice as large as those based on *F*, and *R*- factors based on ALL data will be even larger.

### Fractional atomic coordinates and isotropic or equivalent isotropic displacement parameters (Å^2^)

**Table:**

	*x*	*y*	*z*	*U*_iso_*/*U*_eq_	Occ. (<1)
Cl1	−0.36861 (10)	0.67865 (4)	0.46601 (4)	0.0854 (2)	
Cl2	1.02809 (9)	0.33001 (4)	0.13135 (4)	0.0900 (3)	
N1	0.7137 (3)	0.28257 (9)	0.44997 (10)	0.0559 (5)	
H1N	0.6923	0.2791	0.4897	0.084*	
N2	0.5485 (3)	0.38638 (10)	0.51279 (11)	0.0633 (6)	
N3	0.2373 (3)	0.26039 (11)	0.41612 (11)	0.0681 (6)	
N4	0.0909 (3)	0.36501 (10)	0.48502 (10)	0.0666 (6)	
O1	0.1928 (2)	0.60893 (10)	0.24138 (10)	0.0753 (5)	
H1A	0.2861	0.5960	0.2236	0.113*	
O2	0.3673 (3)	0.63537 (12)	0.32124 (11)	0.0952 (7)	
O3	0.2480 (2)	0.66890 (12)	0.43318 (11)	0.0951 (6)	
H3A	0.3418	0.6628	0.4062	0.143*	
O4	0.2843 (2)	0.47959 (10)	0.15141 (10)	0.0746 (5)	
O5	0.4445 (2)	0.56412 (9)	0.18697 (9)	0.0726 (5)	
C1	0.0776 (3)	0.64335 (12)	0.33999 (13)	0.0547 (6)	
C2	0.0958 (3)	0.66070 (12)	0.40499 (14)	0.0611 (7)	
C3	−0.0418 (3)	0.67074 (13)	0.44405 (13)	0.0626 (7)	
H3	−0.0291	0.6810	0.4874	0.075*	
C4	−0.1972 (3)	0.66515 (12)	0.41731 (13)	0.0594 (7)	
C5	−0.2199 (3)	0.64899 (13)	0.35319 (14)	0.0648 (7)	
H5	−0.3261	0.6457	0.3360	0.078*	
C6	−0.0819 (3)	0.63782 (13)	0.31511 (13)	0.0594 (7)	
H6	−0.0961	0.6264	0.2720	0.071*	
C7	0.2243 (4)	0.62942 (13)	0.30020 (16)	0.0648 (7)	
C8	0.5737 (3)	0.46147 (12)	0.15467 (10)	0.0501 (6)	
C9	0.5603 (3)	0.39317 (13)	0.13526 (12)	0.0595 (6)	
H9	0.4564	0.3747	0.1267	0.071*	0.25
C10	0.7005 (3)	0.35299 (13)	0.12879 (13)	0.0656 (7)	
H10	0.6912	0.3074	0.1168	0.079*	
C11	0.8526 (3)	0.38097 (12)	0.14023 (13)	0.0589 (6)	
C12	0.8724 (3)	0.44813 (13)	0.15866 (12)	0.0569 (6)	
H12	0.9773	0.4663	0.1660	0.068*	
C13	0.7316 (3)	0.48752 (13)	0.16586 (12)	0.0538 (6)	
H13	0.7426	0.5328	0.1786	0.065*	0.75
C14	0.4216 (3)	0.50398 (14)	0.16457 (12)	0.0581 (7)	
C21	0.7978 (4)	0.23170 (13)	0.42364 (13)	0.0662 (7)	
H21	0.8293	0.1946	0.4487	0.079*	
C22	0.8394 (3)	0.23341 (14)	0.35891 (15)	0.0700 (8)	
H22	0.8981	0.1977	0.3403	0.084*	
C23	0.7924 (4)	0.28875 (15)	0.32288 (13)	0.0673 (7)	
H23	0.8182	0.2901	0.2793	0.081*	
C24	0.7056 (3)	0.34341 (12)	0.35095 (12)	0.0560 (6)	
C25	0.6560 (4)	0.40270 (15)	0.31662 (14)	0.0722 (8)	
H25	0.6798	0.4059	0.2730	0.087*	
C26	0.5762 (4)	0.45383 (15)	0.34530 (15)	0.0740 (8)	
H26	0.5459	0.4920	0.3214	0.089*	
C27	0.5366 (3)	0.45098 (12)	0.41213 (14)	0.0618 (7)	
C28	0.4538 (4)	0.50332 (13)	0.44537 (19)	0.0791 (9)	
H28	0.4206	0.5424	0.4236	0.095*	
C29	0.4222 (4)	0.49694 (14)	0.50952 (18)	0.0839 (9)	
H29	0.3688	0.5317	0.5318	0.101*	
C30	0.4707 (4)	0.43793 (14)	0.54105 (15)	0.0779 (8)	
H30	0.4472	0.4342	0.5847	0.093*	
C31	0.5796 (3)	0.39333 (11)	0.44856 (12)	0.0520 (6)	
C32	0.6662 (3)	0.33901 (11)	0.41681 (11)	0.0490 (6)	
C33	0.3118 (4)	0.21255 (15)	0.38143 (17)	0.0835 (10)	
H33	0.3379	0.1717	0.4019	0.100*	
C34	0.3539 (4)	0.21872 (16)	0.31680 (16)	0.0803 (9)	
H34	0.4077	0.1834	0.2954	0.096*	
C35	0.3145 (4)	0.27756 (15)	0.28567 (15)	0.0737 (8)	
H35	0.3409	0.2831	0.2424	0.088*	
C36	0.2336 (3)	0.32990 (13)	0.31938 (12)	0.0565 (6)	
C37	0.1823 (3)	0.39218 (13)	0.28871 (13)	0.0646 (7)	
H37	0.2046	0.3987	0.2453	0.077*	
C38	0.1031 (4)	0.44091 (13)	0.32165 (12)	0.0651 (7)	
H38	0.0716	0.4807	0.3006	0.078*	
C39	0.0659 (3)	0.43319 (11)	0.38807 (11)	0.0537 (6)	
C40	−0.0182 (3)	0.48382 (13)	0.42393 (14)	0.0649 (7)	
H40	−0.0584	0.5227	0.4036	0.078*	
C41	−0.0399 (4)	0.47545 (13)	0.48783 (15)	0.0730 (8)	
H41	−0.0913	0.5091	0.5122	0.088*	
C42	0.0156 (4)	0.41596 (15)	0.51631 (14)	0.0760 (9)	
H42	−0.0005	0.4110	0.5603	0.091*	
C43	0.1165 (3)	0.37377 (11)	0.42085 (11)	0.0522 (6)	
C44	0.1987 (3)	0.31975 (12)	0.38519 (11)	0.0532 (6)	
O6A	0.4152 (3)	0.36325 (13)	0.12245 (13)	0.0825 (8)	0.75
H6A	0.3325	0.3958	0.1317	0.124*	0.75
O6B	0.7525 (8)	0.5525 (3)	0.1865 (4)	0.0664 (19)	0.25
H6B	0.6650	0.5674	0.2002	0.100*	0.25

### Atomic displacement parameters (Å^2^)

**Table:**

	*U*^11^	*U*^22^	*U*^33^	*U*^12^	*U*^13^	*U*^23^
Cl1	0.0676 (4)	0.0996 (5)	0.0890 (6)	−0.0008 (4)	0.0144 (4)	−0.0178 (4)
Cl2	0.0706 (5)	0.0762 (5)	0.1232 (7)	0.0177 (4)	−0.0119 (4)	−0.0287 (5)
N1	0.0713 (13)	0.0502 (11)	0.0463 (12)	0.0009 (10)	0.0083 (11)	0.0075 (9)
N2	0.0744 (16)	0.0533 (12)	0.0623 (15)	−0.0072 (11)	0.0111 (12)	−0.0037 (11)
N3	0.0927 (18)	0.0526 (12)	0.0590 (14)	0.0024 (12)	−0.0153 (13)	0.0074 (11)
N4	0.0941 (18)	0.0588 (12)	0.0469 (14)	−0.0156 (12)	−0.0046 (12)	0.0017 (10)
O1	0.0561 (11)	0.0960 (13)	0.0736 (14)	0.0172 (10)	0.0067 (10)	0.0066 (11)
O2	0.0512 (12)	0.1293 (18)	0.1050 (17)	0.0025 (12)	0.0000 (12)	−0.0156 (14)
O3	0.0612 (13)	0.1030 (15)	0.1212 (18)	0.0034 (12)	−0.0074 (12)	−0.0249 (14)
O4	0.0514 (11)	0.0909 (14)	0.0816 (14)	−0.0026 (10)	−0.0025 (10)	0.0044 (10)
O5	0.0605 (12)	0.0680 (12)	0.0893 (14)	0.0038 (10)	0.0147 (10)	−0.0033 (10)
C1	0.0466 (14)	0.0511 (13)	0.0666 (18)	0.0076 (11)	−0.0001 (13)	0.0036 (12)
C2	0.0502 (16)	0.0534 (14)	0.0796 (19)	−0.0007 (12)	−0.0112 (14)	−0.0017 (13)
C3	0.0628 (18)	0.0589 (14)	0.0660 (17)	−0.0047 (13)	−0.0036 (14)	−0.0064 (13)
C4	0.0600 (16)	0.0477 (13)	0.0705 (19)	0.0023 (12)	0.0074 (14)	−0.0026 (13)
C5	0.0496 (15)	0.0712 (17)	0.074 (2)	0.0037 (13)	−0.0044 (14)	0.0064 (14)
C6	0.0514 (16)	0.0653 (15)	0.0615 (16)	0.0066 (12)	−0.0045 (13)	0.0048 (13)
C7	0.0534 (18)	0.0607 (16)	0.080 (2)	0.0092 (13)	−0.0016 (16)	0.0094 (14)
C8	0.0544 (15)	0.0584 (14)	0.0376 (13)	−0.0047 (12)	0.0013 (11)	0.0080 (10)
C9	0.0557 (16)	0.0701 (16)	0.0525 (16)	−0.0088 (14)	−0.0026 (13)	−0.0023 (13)
C10	0.0682 (18)	0.0590 (15)	0.0695 (18)	−0.0043 (14)	−0.0047 (15)	−0.0086 (13)
C11	0.0594 (16)	0.0622 (15)	0.0551 (16)	0.0072 (13)	−0.0017 (13)	−0.0042 (12)
C12	0.0500 (14)	0.0604 (15)	0.0602 (16)	−0.0021 (12)	−0.0011 (12)	0.0003 (12)
C13	0.0563 (16)	0.0549 (14)	0.0502 (16)	−0.0038 (12)	0.0023 (12)	0.0025 (11)
C14	0.0563 (17)	0.0699 (17)	0.0481 (16)	−0.0007 (14)	0.0084 (13)	0.0161 (13)
C21	0.0786 (19)	0.0538 (15)	0.066 (2)	0.0058 (14)	0.0056 (16)	0.0057 (13)
C22	0.0707 (19)	0.0711 (17)	0.068 (2)	0.0066 (14)	0.0054 (15)	−0.0076 (15)
C23	0.0681 (17)	0.087 (2)	0.0469 (16)	−0.0143 (16)	0.0028 (14)	−0.0019 (14)
C24	0.0586 (15)	0.0609 (15)	0.0486 (16)	−0.0076 (12)	−0.0043 (12)	0.0065 (12)
C25	0.080 (2)	0.084 (2)	0.0525 (17)	−0.0113 (17)	−0.0129 (15)	0.0186 (15)
C26	0.079 (2)	0.0660 (17)	0.077 (2)	−0.0018 (16)	−0.0179 (17)	0.0259 (16)
C27	0.0569 (16)	0.0526 (14)	0.076 (2)	−0.0056 (12)	−0.0137 (14)	0.0085 (14)
C28	0.073 (2)	0.0495 (15)	0.114 (3)	0.0013 (14)	−0.017 (2)	0.0003 (16)
C29	0.084 (2)	0.0571 (17)	0.111 (3)	−0.0002 (16)	0.008 (2)	−0.0194 (18)
C30	0.090 (2)	0.0618 (17)	0.082 (2)	−0.0074 (16)	0.0166 (17)	−0.0185 (16)
C31	0.0497 (14)	0.0463 (12)	0.0598 (17)	−0.0098 (11)	−0.0022 (13)	0.0012 (12)
C32	0.0507 (14)	0.0503 (13)	0.0461 (15)	−0.0090 (11)	−0.0019 (11)	0.0062 (11)
C33	0.107 (3)	0.0593 (17)	0.084 (2)	0.0125 (17)	−0.023 (2)	0.0040 (16)
C34	0.085 (2)	0.0715 (19)	0.084 (2)	0.0115 (16)	−0.0041 (18)	−0.0114 (17)
C35	0.076 (2)	0.0766 (19)	0.0690 (19)	−0.0025 (15)	0.0000 (16)	−0.0024 (16)
C36	0.0614 (15)	0.0573 (15)	0.0508 (16)	−0.0067 (13)	−0.0046 (12)	0.0011 (13)
C37	0.078 (2)	0.0686 (17)	0.0472 (16)	−0.0104 (15)	−0.0025 (14)	0.0102 (13)
C38	0.086 (2)	0.0558 (15)	0.0536 (17)	−0.0028 (14)	−0.0090 (15)	0.0106 (13)
C39	0.0597 (16)	0.0522 (14)	0.0492 (16)	−0.0082 (12)	−0.0088 (12)	0.0023 (12)
C40	0.0722 (18)	0.0515 (14)	0.071 (2)	−0.0046 (13)	−0.0034 (15)	−0.0009 (13)
C41	0.085 (2)	0.0613 (16)	0.072 (2)	−0.0110 (15)	0.0050 (16)	−0.0137 (15)
C42	0.107 (2)	0.0732 (18)	0.0478 (17)	−0.0254 (18)	0.0091 (16)	−0.0070 (15)
C43	0.0641 (16)	0.0487 (13)	0.0437 (15)	−0.0140 (11)	−0.0106 (12)	0.0043 (11)
C44	0.0599 (14)	0.0501 (13)	0.0495 (15)	−0.0065 (12)	−0.0114 (12)	−0.0003 (12)
O6A	0.0541 (15)	0.0888 (17)	0.105 (2)	−0.0090 (14)	−0.0027 (15)	−0.0180 (16)
O6B	0.058 (4)	0.048 (4)	0.093 (5)	−0.006 (3)	−0.001 (4)	−0.022 (4)

### Geometric parameters (Å, °)

**Table:**

Cl1—C4	1.734 (3)	C21—H21	0.9300
Cl2—C11	1.742 (3)	C22—C23	1.372 (4)
N1—C21	1.324 (3)	C22—H22	0.9300
N1—C32	1.359 (3)	C23—C24	1.406 (4)
N1—H1N	0.8464	C23—H23	0.9300
N2—C30	1.327 (3)	C24—C32	1.408 (3)
N2—C31	1.366 (3)	C24—C25	1.421 (4)
N3—C33	1.327 (4)	C25—C26	1.332 (4)
N3—C44	1.365 (3)	C25—H25	0.9300
N4—C42	1.338 (4)	C26—C27	1.427 (4)
N4—C43	1.361 (3)	C26—H26	0.9300
C7—O1	1.312 (3)	C27—C31	1.404 (3)
C7—O2	1.239 (3)	C27—C28	1.405 (4)
O1—H1A	0.8758	C28—C29	1.364 (4)
O3—C2	1.370 (3)	C28—H28	0.9300
O3—H3A	0.9483	C29—C30	1.386 (4)
O4—C14	1.236 (3)	C29—H29	0.9300
O5—C14	1.281 (3)	C30—H30	0.9300
C1—C6	1.391 (3)	C31—C32	1.434 (3)
C1—C2	1.401 (4)	C33—C34	1.391 (4)
C1—C7	1.469 (4)	C33—H33	0.9300
C2—C3	1.389 (4)	C34—C35	1.360 (4)
C3—C4	1.375 (4)	C34—H34	0.9300
C3—H3	0.9300	C35—C36	1.403 (4)
C4—C5	1.382 (4)	C35—H35	0.9300
C5—C6	1.383 (4)	C36—C44	1.411 (3)
C5—H5	0.9300	C36—C37	1.438 (4)
C6—H6	0.9300	C37—C38	1.338 (4)
C8—C13	1.391 (3)	C37—H37	0.9300
C8—C9	1.402 (3)	C38—C39	1.421 (3)
C8—C14	1.496 (3)	C38—H38	0.9300
C9—O6A	1.336 (3)	C39—C43	1.410 (3)
C9—C10	1.384 (3)	C39—C40	1.415 (3)
C9—H9	0.9300	C40—C41	1.350 (4)
C10—C11	1.364 (3)	C40—H40	0.9300
C10—H10	0.9300	C41—C42	1.382 (4)
C11—C12	1.380 (3)	C41—H41	0.9300
C12—C13	1.381 (3)	C42—H42	0.9300
C12—H12	0.9300	C43—C44	1.453 (3)
C13—O6B	1.354 (6)	O6A—H6A	0.9422
C13—H13	0.9300	O6B—H6B	0.8159
C21—C22	1.388 (4)		
			
C21—N1—C32	123.2 (2)	C23—C24—C25	123.7 (3)
C21—N1—H1N	116.6	C32—C24—C25	118.3 (2)
C32—N1—H1N	120.2	C26—C25—C24	121.8 (3)
C30—N2—C31	116.4 (2)	C26—C25—H25	119.1
C33—N3—C44	116.7 (2)	C24—C25—H25	119.1
C42—N4—C43	116.8 (2)	C25—C26—C27	121.0 (3)
C7—O1—H1A	108.4	C25—C26—H26	119.5
C2—O3—H3A	116.5	C27—C26—H26	119.5
C6—C1—C2	118.3 (2)	C31—C27—C28	116.1 (3)
C6—C1—C7	121.4 (3)	C31—C27—C26	120.1 (3)
C2—C1—C7	120.3 (2)	C28—C27—C26	123.8 (3)
O3—C2—C3	116.7 (2)	C29—C28—C27	120.2 (3)
O3—C2—C1	122.3 (3)	C29—C28—H28	119.9
C3—C2—C1	121.0 (2)	C27—C28—H28	119.9
C4—C3—C2	118.7 (3)	C28—C29—C30	119.1 (3)
C4—C3—H3	120.7	C28—C29—H29	120.4
C2—C3—H3	120.7	C30—C29—H29	120.4
C3—C4—C5	122.0 (3)	N2—C30—C29	124.0 (3)
C3—C4—Cl1	118.6 (2)	N2—C30—H30	118.0
C5—C4—Cl1	119.5 (2)	C29—C30—H30	118.0
C4—C5—C6	118.8 (3)	N2—C31—C27	124.2 (2)
C4—C5—H5	120.6	N2—C31—C32	117.7 (2)
C6—C5—H5	120.6	C27—C31—C32	118.0 (2)
C5—C6—C1	121.3 (3)	N1—C32—C24	118.7 (2)
C5—C6—H6	119.4	N1—C32—C31	120.5 (2)
C1—C6—H6	119.4	C24—C32—C31	120.8 (2)
O2—C7—O1	122.6 (3)	N3—C33—C34	125.0 (3)
O2—C7—C1	122.2 (3)	N3—C33—H33	117.5
O1—C7—C1	115.2 (3)	C34—C33—H33	117.5
C13—C8—C9	118.0 (2)	C35—C34—C33	118.4 (3)
C13—C8—C14	121.5 (2)	C35—C34—H34	120.8
C9—C8—C14	120.5 (2)	C33—C34—H34	120.8
O6A—C9—C10	116.5 (2)	C34—C35—C36	119.4 (3)
O6A—C9—C8	122.9 (3)	C34—C35—H35	120.3
C10—C9—C8	120.5 (2)	C36—C35—H35	120.3
C10—C9—H9	119.7	C35—C36—C44	118.3 (2)
C8—C9—H9	119.7	C35—C36—C37	122.2 (2)
C11—C10—C9	119.2 (2)	C44—C36—C37	119.5 (2)
C11—C10—H10	120.4	C38—C37—C36	121.1 (2)
C9—C10—H10	120.4	C38—C37—H37	119.5
C10—C11—C12	122.4 (2)	C36—C37—H37	119.5
C10—C11—Cl2	118.8 (2)	C37—C38—C39	121.5 (2)
C12—C11—Cl2	118.8 (2)	C37—C38—H38	119.3
C11—C12—C13	117.9 (2)	C39—C38—H38	119.3
C11—C12—H12	121.1	C43—C39—C40	117.6 (2)
C13—C12—H12	121.1	C43—C39—C38	119.8 (2)
O6B—C13—C12	117.2 (4)	C40—C39—C38	122.6 (2)
O6B—C13—C8	120.8 (4)	C41—C40—C39	119.7 (3)
C12—C13—C8	121.9 (2)	C41—C40—H40	120.1
C12—C13—H13	119.0	C39—C40—H40	120.1
C8—C13—H13	119.0	C40—C41—C42	118.8 (3)
O4—C14—O5	124.4 (3)	C40—C41—H41	120.6
O4—C14—C8	119.2 (2)	C42—C41—H41	120.6
O5—C14—C8	116.4 (2)	N4—C42—C41	124.6 (3)
N1—C21—C22	120.4 (2)	N4—C42—H42	117.7
N1—C21—H21	119.8	C41—C42—H42	117.7
C22—C21—H21	119.8	N4—C43—C39	122.3 (2)
C23—C22—C21	118.8 (3)	N4—C43—C44	118.5 (2)
C23—C22—H22	120.6	C39—C43—C44	119.2 (2)
C21—C22—H22	120.6	N3—C44—C36	122.1 (2)
C22—C23—C24	120.9 (3)	N3—C44—C43	119.0 (2)
C22—C23—H23	119.6	C36—C44—C43	118.9 (2)
C24—C23—H23	119.6	C9—O6A—H6A	106.4
C23—C24—C32	118.0 (2)	C13—O6B—H6B	109.9

### Hydrogen-bond geometry (Å, °)

**Table:**

*D*—H···*A*	*D*—H	H···*A*	*D*···*A*	*D*—H···*A*
O1—H1A···O5	0.88	1.61	2.484 (2)	173
N1—H1N···N3^i^	0.85	2.14	2.915 (3)	153
O3—H3A···Cl1^ii^	0.95	2.66	3.1714 (19)	114
O3—H3A···O2	0.95	1.86	2.603 (3)	133
O6A—H6A···O4	0.94	1.74	2.584 (3)	148
O6B—H6B···O5	0.82	1.80	2.494 (7)	142
C5—H5···O2^iii^	0.93	2.50	3.404 (3)	164
C12—H12···O4^ii^	0.93	2.51	3.381 (3)	157
C21—H21···N2^i^	0.93	2.51	3.345 (4)	150
C22—H22···O1^iv^	0.93	2.54	3.220 (4)	130
C37—H37···O4	0.93	2.60	3.430 (3)	150
C25—H25···Cg1	0.93	2.65	3.571 (3)	174
C40—H40···Cg2	0.93	2.63	3.489 (3)	154

Symmetry codes: (i) *x*+1/2, −*y*+1/2, −*z*+1; (ii) *x*+1, *y*, *z*; (iii) *x*−1, *y*, *z*; (iv) −*x*+1, *y*−1/2, −*z*+1/2.

## References

[bb1] Altomare, A., Cascarano, G., Giacovazzo, C. & Guagliardi, A. (1993). *J. Appl. Cryst.* **26**, 343–350.

[bb2] Farrugia, L. J. (1997). *J. Appl. Cryst.* **30**, 565.

[bb3] Farrugia, L. J. (1999). *J. Appl. Cryst.* **32**, 837–838.

[bb4] Flack, H. D. (1983). *Acta Cryst.* A**39**, 876–881.

[bb5] Fu, A.-Y., Wang, D.-Q. & Zhang, C.-L. (2005). *Acta Cryst.* E**61**, o3119–o3121.

[bb6] Pan, T.-T., Liu, J.-G. & Xu, D.-J. (2006). *Acta Cryst.* E**62**, m1597–m1599.

[bb7] Rigaku (1998). *PROCESS-AUTO* Rigaku Corporation, Tokyo, Japan.

[bb8] Rigaku/MSC (2002). *CrystalStructure* Rigaku/MSC, The Woodlands, Texas, USA.

[bb9] Sheldrick, G. M. (2008). *Acta Cryst.* A**64**, 112–122. 10.1107/S0108767307043930 18156677

[bb10] Su, J.-R. & Xu, D.-J. (2004). *J. Coord. Chem.* **57**, 223–229.

